# A Neural-Glial Model of the ApoE-SORT1-FABP7 Axis Tied to Sleep Disruption and Alzheimer’s Disease Pathophysiology

**DOI:** 10.3390/biom15101432

**Published:** 2025-10-10

**Authors:** Carlos C. Flores, Yool Lee, Christopher J. Davis, Patrick Solverson, Jason R. Gerstner

**Affiliations:** 1 Translational Medicine and Physiology, Elson S. Floyd College of Medicine, Washington State University, Spokane, WA 99202, USA; carlos.c.flores@wsu.edu (C.C.F.); yool.lee@wsu.edu (Y.L.); cjdavis@wsu.edu (C.J.D.); 2 Sleep and Performance Research Center, Washington State University, Spokane, WA 99202, USA; 3 Steve Gleason Institute for Neuroscience, Washington State University, Spokane, WA 99202, USA; 4 Integrative Physiology and Neuroscience, College of Veterinary Medicine, Washington State University, Spokane, WA 99202, USA; 5 Nutrition and Exercise Physiology, Elson S. Floyd College of Medicine, Washington State University, Spokane, WA 99202, USA; patrick.solverson@wsu.edu; 6 College of Pharmacy and Pharmaceutical Sciences, Washington State University, Spokane, WA 99202, USA; 7 Voiland School of Chemical Engineering and Bioengineering, Washington State University, Pullman, WA 99164, USA

**Keywords:** astrocyte, circadian, metabolism, excitability, neurodegeneration

## Abstract

Alzheimer’s disease (AD) is a complex neurodegenerative disorder where age, genetic factors and sleep disturbance significantly influence disease risk. Recent genome-wide association studies identified a C/T missense variant (rs141749679) in the sortilin (*SORT1*) gene linked to heightened AD risk, revealing SORT1’s role as a key player in the disease’s pathophysiology. This type I membrane glycoprotein is implicated in amyloid β (Aβ) accumulation and associated lipid dysregulation, particularly through its interaction with apolipoprotein E (ApoE). SORT1 facilitates the uptake of ApoE-bound polyunsaturated fatty acids (PUFAs), conversion to endocannabinoids (eCBs), and the regulation of anti-inflammatory pathways via peroxisome proliferator-activated receptors (PPARs). Notably, this neuroprotective signaling is contingent on the *APOE* allele, exhibiting functionality in presence of ApoE3 but disrupted with ApoE4. Additionally, the brain-type fatty acid binding protein, FABP7, mediates this signaling cascade, emphasizing its role in neuron-glia communication. FABP7 is known to regulate sleep across species and binds PUFAs and eCBs. Therefore, dysfunction of the ApoE-SORT1-FABP7 axis may underlie the neuroprotective loss observed in AD, linking sleep disruption and lipid homeostasis to disease progression. This perspective aims to elucidate the intricate neural-glial mechanisms governing the ApoE-SORT1-FABP7 interaction and their implications for targeting therapeutic interventions in Alzheimer’s disease.

## 1. Introduction

Alzheimer’s disease (AD) presents a growing challenge to global health, significantly impacting healthcare systems, economic resources, and social frameworks [1]. As the most common form of dementia, AD affects over 55 million people globally, and this number is expected to triple by 2050 due to an aging population and increased life expectancy [2], which is also influenced by the COVID-19 pandemic [3,4]. The disease is characterized by a gradual decline in cognitive abilities, memory loss, and functional impairments, resulting in higher dependency needs and increased caregiver burdens. The financial impact is substantial, with costs reaching $1.3 trillion annually on a global scale and is expected to rise to US$ 2.8 trillion by 2030, highlighting the urgent need for effective interventions [2]. Despite advancements in understanding the disease’s pathophysiology, including the roles of amyloid β plaques and tau tangles, current treatments remain focused on managing symptoms and modifiable risk factors [5], as there are no available options that can alter the disease’s progression.

AD is a progressive dementia typified by the formation of hyperphosphorylated tau tangles and amyloid plaques in the brain [6,7,8]. It is also associated with sleep disturbances, cognitive decline, memory loss, and other behavioral anomalies [9,10,11]. While AD is not an inevitable part of aging, age is the primary risk factor for its development [12]. Genetic factors also play a significant role, with variations in apolipoprotein E (ApoE) and mutations in the amyloid precursor protein (APP) and presenilin genes (PS1 and PS2) linked to a heightened risk of AD [13,14,15]. The presence of the ApoE4 protein (product of the *APOE ε4* allele) is particularly associated with a higher risk of sporadic late-onset AD. Advances in neuroimaging and biomarkers have prompted a reevaluation of AD, now seen as a progressive neuropathological disease with underlying processes that begin well before clinical symptoms emerge [16,17]. Clinical manifestations can include mild cognitive impairment (MCI), subjective cognitive impairment (SCI), and varying degrees of AD dementia [18,19,20,21,22]. The protracted pre-symptomatic phase of AD pathogenesis presents opportunities for early intervention, which could delay the onset of more severe symptoms and dementia [20,23].

## 2. Alzheimer’s Pathophysiology

### 2.1. A Role for Sleep and Circadian Rhythms

Recent research underscores a strong link between sleep disorders, circadian disruptions, and AD, highlighting their mutual impact on sleep and brain degeneration [10,24,25,26,27,28]. Disrupted sleep patterns, including insomnia, sleep apnea, and fragmented sleep, are both early indicators and risk factors for AD, with poor sleep contributing to Aβ and tau protein accumulation via disturbances in glymphatic clearance during slow-wave sleep. In mammals, circadian rhythms are generated by a cell-autonomous transcriptional feedback loop in which the activators BMAL1 (Brain and Muscle ARNT-Like 1, also known as ARNTL) and CLOCK (Circadian Locomotor Output Cycles Kaput) drive the rhythmic expression of their repressors, PER (Period Circadian Regulator 1–3) and CRY (Cryptochrome Circadian Regulator 1–2). This core oscillatory mechanism is further regulated by an additional complementary loop in which REV-ERBα/β [Nuclear Receptor Subfamily 1 Group D Member 1 and 2 (NR1D1/NR1D2)] and RORα/β [RAR-Related Orphan Receptor Alpha and Beta (RORA/RORB)], act as negative and positive regulators, respectively, of BMAL1 cycling [29,30]. Disruptions of this molecular clock in aging individuals and those with AD exacerbate neurodegeneration by altering physiological activity schedules such as hormone release and brain cell repair [31]. Indeed, BMAL1 and PER1 have been implicated in the production and clearance of Aβ, further connecting circadian dysfunction to AD pathogenesis [32,33,34,35]. Sleep and circadian disruptions accelerate toxic protein buildups and impair cellular repair, thus speeding up neurodegeneration, while neurodegenerative diseases also disturb Sleep–Wake cycles, creating a vicious cycle that worsens disease progression. Understanding the reciprocal relationship between sleep patterns, circadian rhythms, and neurodegeneration is crucial as they both signal and contribute to AD [9,36,37,38,39]. Current research suggests that managing sleep and circadian rhythms could slow neurodegeneration, emphasizing the importance of integrated strategies in research and clinical settings to provide early interventions and improve outcomes for patients with neurodegenerative diseases [36].

### 2.2. A Role for Lipid/Cannabinoid Signaling

FABP7’s role in lipid metabolism in the developing brain is essential, with pronounced expression in embryonic neural stem cells and a sustained presence in astrocytes post-development [40]. FABP7 exhibits a strong binding affinity to ω-3 fatty acids, particularly docosahexaenoic acid (DHA), and plays a critical role in modulating neuroinflammatory processes [41]. Additionally, the ApoE4 variant disrupts sortilin-receptor interaction, thereby muting FABP7 expression, thus qualifying the need for its characterization and investigation in Alzheimer’s pathologies [42,43]. This section presents a brief overview and integration of the present knowledge of the role of FABP7 interactions with PUFAs and cannabinoids, and its plausible role as an interface between ω-3 and endocannabinoid (eCB) systems with downstream implications related to AD progression.

Arachidonic acid (AA) and DHA metabolism in the brain drives pro- and anti-inflammatory signal transduction cascades that play integral roles in neuronal homeostasis, neurogenesis, and inflammation [44]. While both can be made De Novo in the brain from (nutritionally essential) ω-6 and ω-3 precursors, respectively, current evidence suggests a greater reliance on hepatic- or adipose-derived reservoirs of AA and DHA from the blood, and that diet-derived DHA is transported to the brain more effectively than reservoir-derived DHA (see Box 2 of [44]). PUFAs crossing the Blood–Brain Barrier (BBB) will bind FABPs (including FABP7) for transport to storage sites on the plasma membrane and will also bind intracellular FABPs for metabolism to one of several downstream bioactive compounds. DHA is released from glial or neuronal membrane stores Via receptor-mediated signal transduction, or in response to cellular stress, and undergoes metabolism by lipoxygenases to create anti-inflammatory mediators including resolvins, neuroprotectins, and maresin (see Figure 3 of ref. [44]). Freed DHA and its downstream mediators interact directly with multiple cell signaling pathways related to cellular stress. PUFAs also modulate the eCB system, influencing neurotransmitter release Via interaction with cannabinoid receptor type 1 (CB1) of astrocytes, microglia, and neurons. DHA consumption blunts elevated peripheral tissue concentrations of the two primary eCBs, anandamide and 2-arachidonylglycerol (2-AG), in rodents fed a high fat diet [45]. Rodents fed diets devoid of DHA also yield elevated brain 2-AG independent of AA, which could be reversed with dietary replacement of DHA [46]. In addition to direct effects, synaptamide (N-Docosahexaenoylethanolamine) is DHA-derived endocannabinoid that promotes neural growth and function with greater potency than its precursor [47,48] and both brain synaptamide and brain-derived neurotrophic factor (BDNF) concentrations positively respond to dietary intake of DHA [49,50]. While the role of synaptamide in AD is not well known, BDNF depletion has been linked to AD, and may represent a diagnostic marker and target with therapeutic potential for the treatment of AD [51].

Both DHA and its mediator, neuroprotection D1 (NPD1), possess anti-amyloidogenic properties Via inhibition of the enzymatic pathway of amyloid β generation, and DHA is associated with lower plaque formation [52,53,54]. Higher dietary intake of DHA is associated with a lower risk of AD and other neurological conditions [55]. Some but not all brain DHA pools are exhausted in AD, and this is hypothesized to occur in the earlier stages of AD progression [56,57]. However, DHA supplementation does not unequivocally yield positive results in human trials of AD patients [58,59], which could be confounded by *APOE* allele, as ApoE4 reduces DHA uptake into the brain [60,61]. Taken together, the multiple mechanisms of action of DHA underscore a central role in neuronal health, but their effectiveness alone in prevention or reversal of neurodegenerative disease are unproven. This highlights a knowledge gap in the complex interactions between the transport and metabolism of PUFAs, eCB, and *APOE* allele status as they relate to brain health and disease.

### 2.3. Neural-Glial Metabolic Coupling

Growing research has suggested the crucial role of neural-glial metabolic coupling in AD pathophysiology [38,62,63,64]. Neurons and glial cells, especially astrocytes, engage in complex metabolic interactions that maintain brain homeostasis [65]. Disruption of these interactions leads to metabolic dysregulation, compromised neuronal energy supply, and accelerated neurodegeneration, contributing to AD progression [37,66]. Astrocytes are vital for supporting neuronal function by modulating synaptic activity, recycling neurotransmitters, and maintaining the BBB, ensuring energy balance and protection against oxidative stress [67,68,69]. In AD, astrocytes undergo metabolic reprogramming, impairing their neuro-supportive functions [67]. Glial cells, including microglia, also contribute to AD-related inflammation, with metabolic dysregulation triggering chronic inflammation and exacerbating neuronal damage [70,71,72,73]. Recent proteomics studies show a strong link between AD severity and astrocyte-related metabolic proteins [74,75]. Single-nucleus transcriptional profiling of AD patient brains revealed significant metabolic abnormalities in astrocytes, particularly with glutamate Via downregulation of glutamine synthetase (GLUL) and glutamate dehydrogenase 1 (GLUD1), potentially disrupting the glutamate-glutamine cycle and leading to excitotoxicity [76].

Moreover, astrocytes are involved in the clearance of Aβ plaques [77], and dysfunctional astrocytic metabolism impairs Aβ clearance, leading to its accumulation, mitochondrial disruption, and increased neuronal toxicity [78,79]. A recent study revealed that sleep promotes brain energy homeostasis through a neuron-glia mitochondrial lipid cycle, where neurons transfer lipid-linked oxidative damage to glia during the wake cycle, and sleep enables glial lipid clearance, mitochondrial recovery, and neuronal mitophagy [80]. Sleep disturbances, common in AD patients, may exacerbate these metabolic alterations, underscoring the interconnectedness of sleep, metabolism, and neurodegeneration [10,81,82,83].

The implications of glial metabolic dysregulation extend beyond AD, influencing various neurodegenerative conditions such as multiple sclerosis (MS), Parkinson’s disease (PD), and amyotrophic lateral sclerosis (ALS) [84]. Understanding the metabolic coupling between neurons and glial cells is crucial for developing therapeutic strategies aimed at restoring metabolic balance and mitigating neurodegeneration in AD [64,84].

## 3. FABP7: A Molecular Node Integrating Sleep, Circadian Rhythms, Metabolism, and AD

### 3.1. A Role for FABP7 in Sleep and Circadian Rhythms

FABP7 is a member of a family of small, ~15 kD lipid-binding proteins known to bind to hydrophobic regions of fatty acids and their metabolites for transport, influencing a broad spectrum of physiological functions, including PUFAs and their metabolites to facilitate their transport to various subcellular locations. They affect a wide range of cellular processes, including signal transduction, oxidation, membrane synthesis, transcription, fat storage, autocrine/paracrine function, inflammation, and metabolism [85,86]. FABP7 is enriched in astrocytes, oligodendrocyte progenitor cells (OPCs) and neural progenitors and regulates changes in cell growth, morphology, and motility via lipid signaling cascades. We have previously shown that FABP7 expression oscillates in a synchronized fashion throughout the mammalian brain [87,88,89] and is regulated by BMAL1 [90] and REV-ERBa [91]. FABP7 also regulates sleep across phylogenetically diverse species, from flies to mice and humans [92]. Since sleep and circadian disruptions influence neurodegenerative diseases [24,36,37], we sought to determine whether FABP7 plays a role in AD pathophysiology and found that Aβ-induced sleep fragmentation in an Aβ fly model was rescued by overexpression of mouse FABP7 or the fly homolog, dFABP [93]. Overexpression of FABP7 or dFABP in flies was also shown to promote long-term memory formation and sleep consolidation [94], and nuclear-cytoplasmic localization seems to correlate with different forms of memory [95]. Given that circadian factors are known to contribute to long-term memory formation and synaptic plasticity [96,97], FABP7 may function as a molecular node that integrates astrocyte lipid metabolism with sleep, clocks, and cognitive function [91]. Taken together, these studies indicate that FABP7 and related neural-glial signaling cascades likely cooperate with circadian/Sleep–Wake states to drive lipid signaling, metabolism, and neuroprotection [37,38,91,98].

### 3.2. A Role for FABP7 in Neural-Glial Metabolic Coupling in AD

Recent studies suggest that FABP7 is essential for maintaining neuronal structure and synaptic function through mediating neuron-glia metabolic coupling [99,100]. FABP7 is predominantly expressed in astrocytes and OPCs, but not in mature neurons or microglia. FABP7 knockout (KO) mice exhibit reduced dendritic arborization and impaired synaptic plasticity [99]. Specifically, pyramidal neurons in the medial prefrontal cortex (mPFC) show decreased dendritic branching, shorter arbor length, lower synapse density, and weakened excitatory synaptic transmission [99]. Similarly, wild-type neurons co-cultured with FABP7-deficient astrocytes show reduced dendritic complexity and spine density, confirming the crucial role of astrocytic FABP7 in supporting neuronal maturation [99]. Furthermore, recent research reveals that FABP7, along with its family members FABP3 and FABP5, bind neuroactive lipids such as epoxyeicosatrienoic acids (EETs) and 15-deoxy-Δ12,14-prostaglandin J2 (15d-PGJ2), regulating EET-mediated synaptic signaling and emphasizing their broader role in neuronal lipid signaling [101]. Together, these findings highlight the importance of FABP7-mediated neuron-glia lipid metabolism in synaptogenesis, supporting neural homeostasis and function [100].

Reflecting its critical role in neural physiology and function, growing evidence suggests that FABP7 is implicated in the pathophysiology of neurological and neurodegenerative diseases, particularly AD [98,102,103]. In AD, FABP7 expression is upregulated in astrocytes around amyloid plaques, while ApoE4 disrupts myelin homeostasis in the frontal cortex by altering ApoE and FABP7, contributing to early demyelination and cognitive decline [104]. This upregulation is associated with an inflammatory response, as FABP7 overexpression in astrocytes induces NF-κB-driven inflammation and neurotoxicity [104,105]. Proteomics studies revealed that compared to asymptomatic AD brain, significantly elevated levels of FABP7 in AD brain were observed [74,75,106,107]. Notably, FABP7 binds to both AA and DHA [41], resulting in distinct physiological responses [98,108]. The binding of AA to FABP7 is thought to promote inflammatory pathways and astrogliosis, which can impede glutamatergic uptake and exacerbate neuroinflammation [98,105,108,109], while the binding of DHA to FABP7 conversely stabilizes astrocyte-neuron lactate shuttle dynamics, preserves glutamatergic uptake, and activates anti-inflammatory pathways, promoting neuroprotection [91,98,105,108]. The FABP7’s dual role in AD makes it a potential therapeutic target to restore metabolic balance and reduce neuroinflammation, warranting further research to develop targeted interventions.

### 3.3. A Role for FABP7 and Cannabinoids Beyond DHA Signaling in AD

In addition to shuttling DHA, FABP7 transports the eCB anandamide and the two predominant cannabinoids sourced from *Cannabis sativa*, delta 9-Tetrahydrocannabinol (THC) and the non-psychoactive Cannabidiol (CBD) [110,111]. Recently, THC-treated FABP7 KO mice were shown to increase distance travelled in an open field, the opposite phenotype of WT THC-treated mice, which reduced distance traveled [112], suggesting a regulatory role for FABP7 mediating the effects of THC on behavior. Both PUFA and cannabinoid-based interventions are proven modulators of endocannabinoid system tone (ECS) via modulation of CB1, which is demonstrated to inhibit neuroinflammatory pathways [113]. Indeed, the observed changes in ECS tone translate to clinical improvements. For example, a meta-analysis of four separate Phase III clinical trials included 550 epileptics and reported a 20% reduction in seizure frequency in patients consuming 10–20 mg/kg body weight CBD for up to 14 weeks [114]. Elucidation of the mechanistic underpinnings between the brain’s eicosanoid and endocannabinoid systems, including a greater framework for the essential role(s) of FABP7 within and between both, will inform its influence on both metabolic and oxidative stress conditions, and how the axis can be exploited to improve clinical outcomes of neurological disease. To this last point, FABP7 and other brain FABP isoforms were recently implicated in facilitation of the interaction of AA-derived EETs, which promote PPAR-gamma activity, with downstream effects on synaptic transmission and other critical functions [101]. Enhancement of these signaling pathways attenuates AD progression in 5xFAD mice [115,116]. In conclusion, given FABP7’s dual role in transporting both DHA-derived mediators and cannabinoids, it emerges as a novel therapeutic target bridging dietary fatty acid interventions and modulation of cannabinoid signaling pathways to mitigate the neuroinflammation of AD.

## 4. ApoE-SORT1-FABP7 Axis in AD

**Figure 1:**
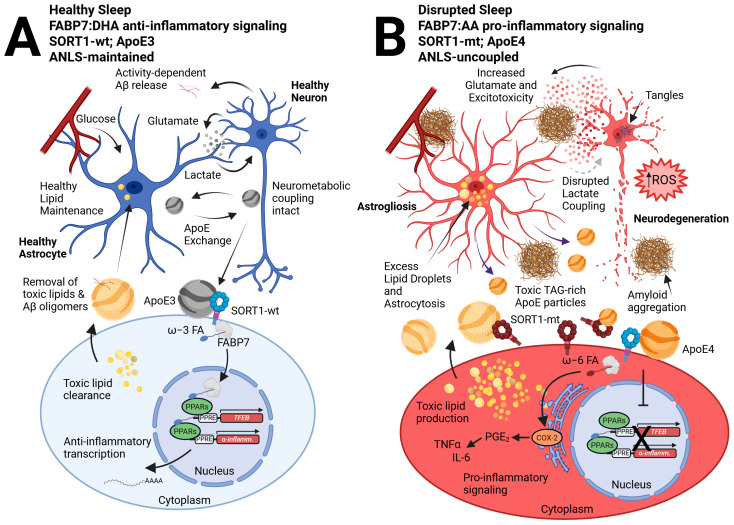
A neural-glial model of the ApoE-SORT1-FABP7 axis tied to sleep disruption and AD pathophysiology. We previously described how neural-glial metabolic coupling during healthy sleep preserves neural function via the astrocyte supply of lactate following activity-dependent glutamate release tied to wakefulness, known as the ANLS [62,124]. (**A**) In contrast, disrupted sleep is tied to enhanced wake-dependent glutamate release from neurons, subsequent Aβ oligomerization, and astrocyte reactivity, creating a reciprocal interaction that disrupts the ANLS and enhances glutamatergic release, which is tied to further fragmented sleep and excitotoxicity that perpetuates the cycle (**B**) [37,38]. In this updated model, we incorporate FABP7 signaling from astrocytes to neurons that is closely tied to ApoE lipid exchange, where ApoE3 cargo interacts with SORT1-wt receptors on neurons to drive FABP7-dependent ω-3 FA, such as DHA nuclear localization, to promote PPAR-mediated transcription of anti-inflammatory and neuroprotective genes, such as *TFEB* (**A**). However, ApoE4 disrupts functional interactions between SORT1 and FABP7 (**B**), preventing nuclear localization of FABP7 and subsequent neuroprotective gene expression [42,123]. We hypothesize that the recently identified SORT-mt tied to Alzheimer’s risk, in combination with the *Ex17b* mutant that permits soluble decoy SORT1 receptors [125,126], may similarly inhibit the ApoE-SORT1-FABP7 nuclear lipid-signaling cascade. This would favor FABP7 to bind to ω-6 FAs, such as AA, and shuttle them to the endoplasmic reticulum to activate COX-2-dependent pro-inflammatory pathways via ω-6 FA conversion to prostaglandins (particularly PGE_2_). Such activation would result in the production of chemokines and cytokines, such as TNFα and IL-6, in tandem with our previous hypothesis for a dichotomous role for FABP7 in AD [98]. Here, pro-inflammatory signaling further exacerbates toxic lipid production via increased glutamate release, excitotoxicity, and ROS leading to excess lipid droplet formation and astrocytosis, disrupting lactate coupling. Further release of toxic TAG ApoE particles as well as enhanced amyloid secretion and aggregation tied to more glutamate leads to neurodegeneration. We hypothesize that the wake-dependent Aβ and TAG release by neurons is taken up by ApoE and trafficked to astrocytes for clearance and metabolism as a process during sleep. However, given SORT1 expression is not exclusive to neurons, future studies will be important to distinguish between cell types responsible for ApoE particle trafficking, ligand uptake, intracellular routing, nuclear signaling, and secretion events. Abbreviations: astrocyte-neuron lactate shuttle (ANLS), beta-amyloid (Aβ), wild-type (wt), omega-3 (ω-3), fatty acid (FA), docosahexaenoic acid (DHA), peroxisome proliferator-activated receptor (PPAR), transcription factor EB (*TFEB*), mutant (mt), omega-6 (ω-6), arachidonic acid (AA), cyclooxygenase 2 (COX-2), prostaglandins E2 (PGE_2_), tumor necrosis factor-alpha (TNFα), interleukin-6 (IL-6), reactive oxygen species (ROS), triacylglycerol (TAG).

A newly discovered C/T missense variant, rs141749679, of the gene for the membrane surface receptor sortilin (*SORT1*) was recently shown to be associated with increased AD risk in genome-wide association studies (GWAS) [117]. SORT1 is a type I membrane glycoprotein in the vacuolar protein sorting 10 protein (VPS10P) family of sorting receptors that also includes SorLA, SorCS1, SorCS2, and SorCS3, all of which have been identified as AD risk loci [118]. While SORT1 is expressed in neurons, it is not exclusive to this cell type, as it has also been shown to be expressed in various glial cells, including astrocytes, oligodendrocytes, OPCs, and microglia, as well as endothelial cells in humans and mice [119,120]. Existing mouse models with targeted *Sort1* gene disruption have shown increased levels of Aβ peptides and plaque burden in the brain, as well as the accumulation of sulfatides (a type of glycolipid) likely associated with ApoE-Aβ complex dysfunction [121]. Given that SORT1 binds to ApoE with high affinity, it is probable that the dysregulation of lipid homeostasis that contributes to AD pathology may be due in part to functional deficits of ApoE-SORT1 interactions [122]. Indeed, SORT1 directs the uptake of ApoE-bound PUFAs and their conversion into eCBs, and the regulation of anti-inflammatory gene expression programs via the peroxisome proliferator-activated receptor (PPAR) family of transcription factors in an *APOE* allele-dependent manner [123]. The neuroprotective effects of SORT1 exist with functional ApoE3, but are disrupted upon ApoE4 binding [123]. Unbiased proteome screens have discovered that this *APOE*-allele-dependent neuroprotective signaling is mediated by functional expression of FABP7. In the presence of ApoE3, a SORT1-FABP7 signaling cascade elicits stimulation of PPAR-mediated gene expression, which is blocked in the presence of ApoE4 [42]. Therefore, dysfunctional ApoE-SORT1-FABP7 neural-glial signaling may contribute to the loss of neuroprotection observed in AD (Figure 1).

## 5. Conclusions

The manuscript highlights the critical role of the ApoE-SORT1-FABP7 axis in the complex neurodegenerative processes underlying Alzheimer’s disease. Disruptions in this pathway—particularly stemming from the ApoE4—impair lipid metabolism, neuroinflammatory regulation, and neuroprotective signaling mediated by endocannabinoids and PPARs. The interaction between these molecular components influences sleep regulation, neuronal-glial metabolic coupling, and amyloid pathology, thereby contributing to disease progression. Determining the relationship between FABP7 and sleep with AD progression remains an important area of future study. Understanding this axis offers promising avenues for targeted therapeutic interventions aimed at restoring lipid homeostasis, reducing neuroinflammation, and mitigating sleep disruptions. Overall, elucidating the neural-glial mechanisms governing the ApoE-SORT1-FABP7 pathway enhances our comprehension of AD pathophysiology and opens potential strategies to delay or prevent neurodegeneration in at-risk populations.

## Data Availability

Not applicable.
